# Detecting Selection Using Time-Series Data of Allele Frequencies with Multiple Independent Reference Loci

**DOI:** 10.1534/g3.113.008276

**Published:** 2013-09-30

**Authors:** Jo Nishino

**Affiliations:** Center for Information Biology and DNA Data Bank of Japan, National Institute of Genetics, Research Organization of Information and Systems, Mishima, Shizuoka 411-8540, Japan

**Keywords:** selection, neutrality, time-series data of allele frequencies, reference loci, population size fluctuations

## Abstract

Recently, in 2013 Feder *et al.* proposed the frequency increment test (FIT), which evaluates natural selection at a single diallelic locus by the use of time-series data of allele frequencies. This test is unbiased under conditions of constant population size and no sampling noise. Here, we expand upon the FIT by introducing a test that explicitly allows for changes in population size by using information from independent reference loci. Various demographic models suggest that our proposed test is unbiased irrespective of fluctuations in population size when sampling noise can be ignored and that it has greater power to detect selection than the FIT if sufficient reference loci are used.

In population genetics, most data are obtained from a single point in time. When genetic time-series data are available, the use of such data to detect and estimate natural selection is an attractive concept. Time-series data may have direct information about natural selection because they affect allele frequencies in time. Bollback *et al.* (2008) introduced a statistical framework for estimating and testing natural selection by using time-series data of allele frequencies at a single diallelic locus. These authors applied their framework to an ancient human DNA sequence (Hummel *et al.* 2005) and a sample from an experimental evolution study of the bacteriophage, MS2 (Bollback and Huelsenbeck 2007).

Recent advances in high-throughput sequencing technology, including pooled DNA sequencing, have facilitated the acquisition of time-series data, and Bollback *et al.*’s (2008) method has been extended to more complicated situations. Mathieson and McVean (2013) applied a lattice model of population subdivision that enabled joint estimation of migration rate and spatially varying selection coefficients. The allele age also is an important parameter in selection inferences because it can provide information regarding the origin of a particular phenotype associated with the allele. Malaspinas *et al.* (2012) developed a method to estimate the selection coefficient and the allele age simultaneously. In addition, there has been increasing numbers of studies in which researchers focus on specific settings in each evolutionary experiment (*e.g.*, Illingworth and Mustonen 2011; Gallet *et al.* 2012; Illingworth *et al.* 2012).

Feder *et al.* (2013) reported recently that Bollback *et al.*’s (2008) standard χ^2^-based test for selection is biased for realistic data with few sampled time points. When the number of sampled time points is sufficiently large, the likelihood ratio statistic (LRS) follows a χ^2^ distribution. However, the actual number of sampled time points rarely exceeds a few dozen. Particularly, when the null hypothesis is composite and the profile likelihood is used, the estimation of nuisance parameters can substantially bias inferences of the parameters of interest (*e.g.*, see Chapter 10 of Pawitan 2001). For the problem described in this report, the nuisance parameter is the population size.

To avoid bias, Feder *et al.* (2013) proposed two methods that both were modeled under conditions of constant population size and no sampling noise. In the empirical likelihood ratio test (ELRT), the population size is preliminarily estimated under neutrality as a first approximation. The estimated population size then is used to generate the empirical distribution of the LRS by computer simulation. Neutrality then can be evaluated by comparing the observed LRS with the empirical distribution. Although the ELRT was shown to be unbiased, this approach can be computationally intensive. To reduce the computational load, Feder *et al.* (2013) proposed the frequency increment test (FIT). The statistic used for FIT is defined as the following: Let $x_{0},\;x_{1},\ldots ,x_{L}$ be the population frequencies of one allele at a diallelic locus of interest at the sampled time, $t_{0}=0,\;t_{1},\ldots ,t_{L}$. The sampling time scales are short compared to the population size. Then the standardized allele frequency increment,

$$Y_{i}=\frac{x_{i}-x_{i-1}}{\sqrt{2x_{i-1}\left(1-x_{i-1}\right)\left(t_{i}-t_{i-1}\right)}},\;i=1,2,\ldots ,L,\;$$ (1)

is approximately normally distributed with mean 0 under the null model, that is, neutral evolution. The variance of *Y_i_* is equal to 1/(2*N*) in the Wright−Fisher model with *N* diploids. However, the variance is unknown because *N* is unknown. In such a situation, natural selection can be evaluated by letting

$$t_{FIT}\left(Data\right)=\frac{\bar{Y}}{\sqrt{S^{2}/L}},$$

where $\bar{Y}$ and *S* are the sample mean and variance of *Y_i_*, respectively. We then perform a *t*-test using the fact that *t_FIT_* follows the Student’s *t* distribution with *L* − 1 degrees of freedom under the null model. This is the FIT.

The FIT treats the nuisance parameter, *N*, as an unknown parameter instead of estimating it. When *Y_i_* for any *i* follows the normal distribution with the same variance under the null model, the FIT is an exact and unbiased test. Feder *et al.* (2013) verified that actual type I error rates approach the nominal significance level for various parameter settings. These investigators also demonstrated that the power of the FIT is equal to or greater than that of the ELRT. Although the FIT does not account for the sampling process from a population explicitly, the test was shown to work well even when the sampling process exists if the sample size is not small.

The FIT is a simple and bias-controlled method to detect selection. However, it is not clear whether the FIT works well when the population size fluctuates. Theoretically, under the null model with fluctuating population size *Y_i_* does not follow the same normal distribution for all *i*, and therefore, *t_FIT_* (Data) does not follow the Student’s *t* distribution.

This study is an extension of Feder *et al.*’s (2013) FIT that allows for fluctuations in population size by using reference loci. First, the FIT’s actual type I error rates in a fluctuating population is investigated. Then, a new test is introduced, the frequency increment test with reference loci (FITR). Given a fluctuating population size, the FITR’s actual type I error rates are almost the same as the nominal significance level. Then, the powers of the FITR and the FIT to detect natural selection were evaluated. Finally, the simulation method used in this study was validated and the properties of the FITR in practical situations were investigated. Model descriptions are presented just below and added before introducing the FITR.

## Materials and Methods

### Model and simulation methods

**Figure 1:**
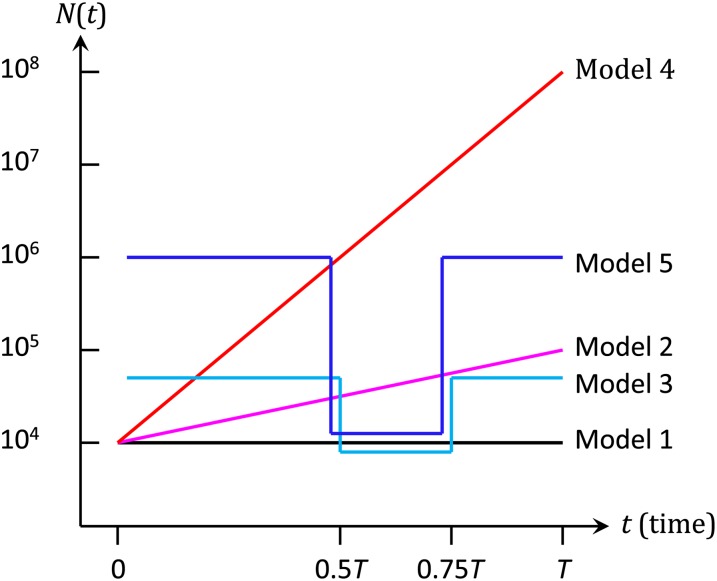
Demographic models used in this study. Model 1: constant-size model (*N*(*t*) = 10^4^); Model 2: slow-growth (grows exponentially from *N*(0) = 10^4^ to *N*(*T*) = 10^5^); Model 3: moderate-bottleneck model ($N\left(0\leq t<0.5T\right)=N\left(0.75\leq t\leq T\right)=5\times 10^{4}\text{ and }N\left(0.5\leq t<0.75T\right)=\;10^{4}$); Model 4: rapid-growth model ($\text{grows exponentially from}N(0)=10^{4}\text{ to }N(T)=10^{8}$); Model 5: severe-bottleneck model ($N\left(0\leq t<0.5T\right)=N\left(0.75\leq t\leq T\right)=10^{6}\text{ and }N\left(0.5\leq t<0.75T\right)=\;10^{4}$).

Let us consider a population evolving according to the Wright–Fisher model with fluctuating population size. The population size fluctuates as a function of generation time, *t*, and is denoted by *N*(*t*). To investigate the actual type I errors and the powers of Feder *et al.*’s (2013) FIT and the FITR introduced in this study, we conducted computer simulations under the five demographic models shown in Figure 1. The two alleles at a diallelic locus of interest are denoted by *A*_0_ and *a*_0_, respectively. At generation times $t_{0}=0,\;t_{1},\ldots ,t_{L}=T$, the frequencies of *a*_0_ are denoted by $x_{0,0},\;x_{0,1},\;\ldots ,x_{0,L}$. Here, *t*_0_ = 0 and *t_L_* = *T* are the first and the last sampling times, respectively, and the number of sampled times is *L* + 1. The fitnesses of genotypes *A*_0_*A*_0_, *A*_0_*a*_0_, and *a*_0_*a*_0_ are assumed to be 1, $1+0.5s_{0}$, and $1+s_{0}$, respectively (*i.e.*, no dominance is assumed). The population size, *N*(*t*), is independent of the frequency of *a*_0_. As described in the next section, the FITR also uses neutral reference loci.

In the Wright–Fisher model, the allele frequency can be obtained exactly every generation as a binomial distribution. However, the generation of these data poses an extreme computational burden that is impractical for large populations (Figure 1). To avoid this burden and simulate changes in allele frequencies, we applied the pseudo-sampling method (Kimura and Takahata 1983), which is an improved version of the methods of Kimura (1980). In this method, to determine the allele frequency every generation, a uniform random number with the same mean and variance as those of the exact binomial distribution is used when the allele frequency is moderate. When the allele frequency is high or low, a Poisson random number with the same mean as that of the exact binomial random number is used. In this study, a frequency of ≤5 minor alleles in the population was used as the criteria for high or low allele frequencies. In addition, the normal distribution was used instead of the uniform distribution because the normal distribution better approximates the binomial distribution, which next-generation allele frequencies follow under the Wright–Fisher model.

## Results and Discussion

### Type I error rate of FIT

Table 1 summarizes the actual type I error rates for Feder *et al.*’s (2013) FIT. In demographic model 1 (constant-size model), as shown by Feder *et al.* (2013), the actual type I error rates approach the nominal level. For model 2 (slow-growth model) and model 3 (moderate-bottleneck model), the test becomes somewhat conservative. For the purposes of controlling type I error, this is a desirable property. However, in model 4 (rapid-growth model) and model 5 (severe-bottleneck model), the test tends to be too conservative, causing it to have less power to detect selection when the population size is fluctuating.

**Table 1:** Actual type I error rates (%) of FIT

*T*	*L*	Δ*t**^a^*	Model 1	Model 2	Model 3	Model 4	Model 5
10	2	5	4.99	4.33	4.20	1.05	1.30
100	20	5	4.96	4.81	4.70	3.32	3.42
1000	200	5	4.89	4.90	4.97	4.87	4.87
10	5	2	4.96	4.23	3.99	0.48	0.63
100	5	20	4.90	4.03	4.06	0.44	0.68
1000	5	200	5.30	4.17	4.36	0.49	0.72

Values indicate the actual type I error rates obtained by 100,000 simulations under a nominal significance level of 5%. The initial allele frequency, *x_0,0_*, was assumed to be 0.5. FIT, frequency increment test.

a $\Delta t=t_{i}-t_{i-1}\;\left(i=1,2,\ldots ,L\right)$.

### Frequency increment test with reference loci (FITR)

Here we propose a new test, the FITR. Consider *R* reference loci in addition to the focal locus. It is assumed that these R+1 loci are evolving independently and that *R* reference loci are evolving under neutrality. We denote by $x_{h,0},\;x_{h,1},\ldots ,x_{h,L}\;\left(h=1,2,\ldots ,R\right)$ the population frequencies of one allele at the *h*-th reference diallelic locus at times $t_{0}=0,\;t_{1},\ldots ,t_{L}=T$. Recall that *x*_0*,i*_ is the allele frequency of the focal locus at *t_i_*. Suppose that *N*(*t*) is a step function and let *N_i_* be the population size from $t_{i-1}$ to $t_{i}$ such that $N\left(t_{i-1}<t\leq t_{i}\right)=N_{i}$. Note that although the following FITR discussion assumes N(t) is a step function, the same discussion can apply even the case that *N*(*t*) is a continuous function (Figure 1) because *N_i_* can be interpreted as the variance effective size over the period from $t_{i-1}\text{ to }t_{i}$. For this reason, the FITR is unbiased irrespective of fluctuations in population size.

When $x_{h,i-1}$ is not close to 0 or 1 and $\Delta t_{i}=t_{i}-t_{i-1}$(i=1,2,…,L) is small compared with *N_i_*,

$$Y_{h,i}=\frac{\Delta x_{h,i}}{\sqrt{\frac{\Delta t_{i}}{2N_{i}}}},$$ (2)

where

$$\Delta x_{h,i}=\frac{x_{h,i}-x_{h,i-1}}{\sqrt{x_{h,i-1}\left(1-x_{h,i-1}\right)}},$$

follows the standard normal distribution for *h* = 0 under the null model and for h=1, 2, …,R under the null or alternative models. We then consider whether the allele frequency change from $t_{i-1}\;\text{to}\;t_{i}$ for the focal locus, *Y*_0*,i*_, is significant. If *N_i_* is known, we can test for neutrality using the fact that *Y*_0*,i*_ follows the standard normal distribution. In this case, however, *N_i_* is unknown. Let us then define a statistic,

$$t_{FITR(i)}\left(Data\;\text{from}\;t_{i-1}\;\text{to}\;t_{i}\right)=\frac{Y_{0,i}}{\sqrt{\frac{1}{R}\sum_{h=1}^{R}Y_{h,i}^{2}}}$$ (3)

$$=\frac{\Delta x_{0,i}}{\sqrt{\frac{1}{R}\sum_{h=1}^{R}\Delta x_{h,i}^{2}}}.$$ (4)

The statistic $t_{FITR\left(i\right)}$ is independent of *N_i_*, as seen in (4), because *N_i_* in (3) is canceled out. In addition, $t_{FITR\left(i\right)}$ is independent of Δ*t_i_*. In (3), the numerator follows the standard normal distribution, and the denominator is equal to the square root of the χ^2^ random variable divided by its degrees of freedom, *R*. Because the numerator and denominator are independent, $t_{FITR(i)}$ follows a Student’s *t* distribution with *R* degrees of freedom (Fisher 1925). Although we determined the form of the test statistic, $t_{FITR(i)}$, intuitively, $t_{FITR\left(i\right)}$ can be derived as the exact LRS using data, $\Delta x_{i}=(\Delta x_{0,i},\Delta x_{1,i},\ldots ,\Delta x_{R,i})$, in a plausible setting (see Appendix). In other words, the aforementioned *t*-test is equivalent to the likelihood ratio test under conditions of normality, as observed in several statistical situations (see, *e.g.*, Lehmann and Romano 2005).

Next, let us define a statistic using all the data from $t_{0}=0$ to $t_{L}=T$, $\Delta x=(\Delta x_{0},\Delta x_{1},\ldots ,\Delta x_{L})$,

$$t_{FITR}\left(Data\right)=\frac{1}{\sqrt{L}}\sum_{i=1}^{L}t_{FITR(i)}\;$$ (5)

$$=\sum_{i=1}^{L}\frac{\Delta x_{0,i}}{\sqrt{\frac{L}{R}\sum_{h=1}^{R}\Delta x_{h,i}^{2}}},$$ (6)

$t_{FITR}$ is the standardized sum of $t_{FITR(i)}$ overall *i*. The standardization factor $1/\sqrt{L}$ allows for a straightforward interpretation of the statistic because $t_{FITR}$ asymptotically follows the standard normal distribution as *R* becomes large. The exact distribution of $t_{FITR}$ with infinite *R* is difficult to express explicitly, but the distribution of $t_{FITR}$ can be obtained empirically by generating *L* Student’s *t* random variables with *R* degrees of freedom and summing them. This approach is valid because each $t_{FITR(i)}$ follows a Student’s *t* distribution with *R* degrees of freedom. We obtained $t_{FITR}$ using the statistical package R (http://www.R-project.org) with 100,000 simulations of $t_{FITR}$ for each combination of *R* and *L*. The test using $t_{FITR(i)}$ is the FITR, an exact significance test assuming *Y_h,i_* follows the standard normal distribution. That is, the actual type I error rate of the test is expected to be close to the nominal significance level regardless of fluctuations in population size. Unlike $t_{FITR(i)}$, the $t_{FITR}$ statistic is not the exact LRS, which is very difficult to express explicitly. An ad hoc interpretation of the test statistic, *t_FITR_*, is presented in the Appendix.

### Type I error rate of FITR and powers of FITR and FIT

**Figure 2:**
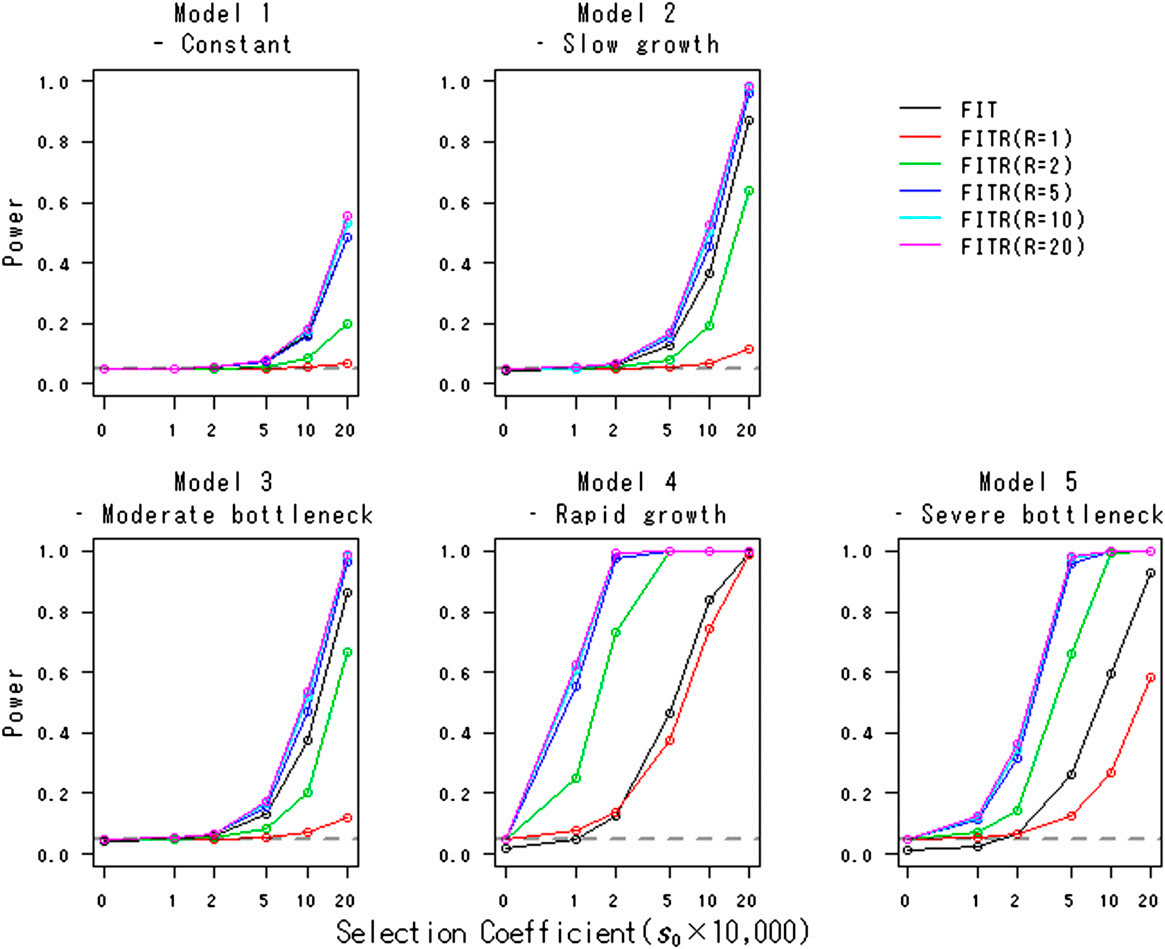
Powers of the FITR and the FIT in various demographic models. Powers of the FIT (black line) and the FITR with *R* reference loci (colored lines) are shown as functions of the selection coefficients in the five demographic models. Each point corresponds to the power obtained by 100,000 simulations at the 5% significance level. The duration of sampling time and the number of sampled points were *T* = 1000 and (*L* + 1) = 11, respectively. The intervals between any two adjacent sampled points were the same at $\Delta t=t_{i}-t_{i-1}=100\;(i=1,2,\ldots ,L)$. The initial frequency for all *R*+ 1 loci, $x_{h,0}$, was assumed to be 0.5.

Table 2 shows the actual type I error rates of the FITR. As expected, for all demographic models, the actual type I error rates are close to the nominal level. Figure 2 shows the powers of the FITR and the FIT as a function of the strength of selection. In all demographic models, including the constant-size model, the FITR had more power than the FIT if five or more reference loci were used. For model 2 (slow-growth model) and model 3 (moderate-bottleneck model), the power of the FIT was acceptable. However, for model 4 (rapid-growth model) and model 5 (severe-bottleneck model), the power of the FIT was relatively small, and the FITR demonstrated much larger power than the FIT.

**Table 2:** Actual type I error rates (%) of FITR

*T*	*L*	Δ*t**^a^*	Model 1 (*R* = 5)*^b^*	Model 2 (*R* = 2)	Model 3 (*R* = 20)	Model 4 (*R* = 1)	Model 5 (*R* = 10)
10	2	5	4.99	5.02	4.95	5.12	5.00
100	20	5	5.09	4.95	5.06	5.05	5.00
1000	200	5	5.07	4.95	5.01	5.02	5.24
10	5	2	4.98	4.94	5.06	4.95	4.94
100	5	20	4.98	5.17	5.20	4.90	5.02
1000	5	200	4.87	5.02	5.06	4.89	5.02

Values indicate the actual type I error rates obtained by 100,000 simulations under a nominal significance level of 5%. The initial frequencies for all *R* +1 loci, *x_h_*_,0_, are assumed to be 0.5. FITR, frequency increment test with reference loci.

a $\Delta t=t_{i}-t_{i-1}\;\left(i=1,2,\ldots ,L\right)$.

b The numbers of reference loci, *R*, are randomly assigned to each demographic model.

**Figure 3:**
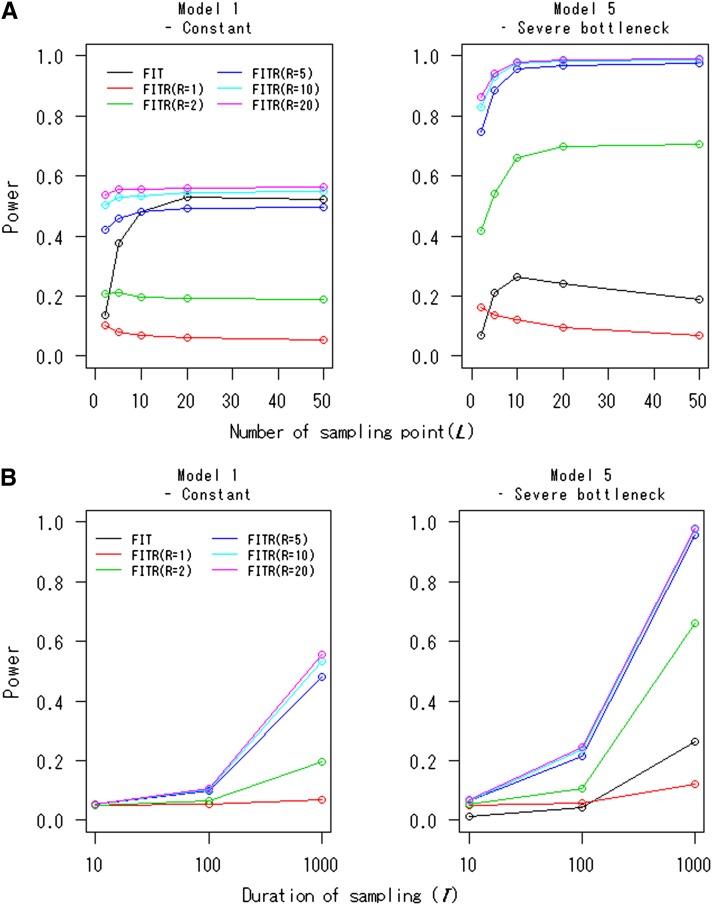
Powers of the FITR and the FIT as functions of (A) the number of sampling points and (B) the duration of sampling. Powers of the FIT (black line) and the FITR with *R* reference loci (colored lines) are shown for the demographic models 1 and 5. Each point corresponds to the power obtained by 100,000 simulations at the 5% significance level. The selection coefficients were *s* = 0.002 for model 1 and *s* = 0.0005 for model 5. The intervals between any two adjacent sampled points were the same, $\Delta t=t_{i}-t_{i-1}=T/L\;(i=1,2,\ldots ,L)$. The initial frequency for all *R*+1 loci, $x_{h,0}$, was assumed to be 0.5. (A) The duration of sampling time was fixed at *T* = 1000. (B) The number of sampled points was fixed at *L* = 10. (Note: The FIT curve nearly overlapped with the FITR curve for *L* = 5 in Model 1.)

Figure 3A displays the powers of the FITR and the FIT as functions of the number of sampling points, *L*. In many cases, the powers approached certain asymptotic values with increasing *L*. In this case, the values of *L* for which the powers approached their asymptotes were relatively small (*e.g.*, *L* = 10 or 20). The powers of the FITR for *R* = 1 or 2 in Model 1 decreased somewhat as the sampling points increased. This trend also was observed for the FITR with *R* = 1 and for the FIT in Model 5. The powers of the FITR and the FIT as functions of the duration of sampling time, *T*, are given in Figure 3B. For all cases, the powers increased with increasing *T*, as expected. For Figure 3, A and B, the FITR had more power than the FIT if 10 or more reference loci were used even in the constant-size model. This difference in power was more obvious for model 5 (severe-bottleneck model).

### Applying the simulation method and FITR to practical situations

The FITR was developed and evaluated for type I error rate and power under ideal conditions of “selectively neutral” reference loci evolving independently of the focal locus and of each other, allele frequencies at reference loci ≠ 0 or 1, definable FITR statistics, and exactly known population allele frequencies. In practice, these ideal conditions may be violated. Before this section, the computer simulation method used in this study had not been validated. Here, we discuss the applicability of the simulation and describe cases that violate the aforementioned assumptions.

For the determination of allele frequencies in successive generations at *R* + 1 loci, the exact binomial sampling is computationally intensive and impractical for realistic population sizes (Figure 1). Therefore, we used a pseudosampling method to simulate the binomial sampling process (Wright−Fisher model). Even for a small, constant-size (*n* = 20) Wright−Fisher population, the fixation times for a mutant obtained by the pseudosampling were consistent with those obtained by binomial sampling (Kimura 1980). However, we considered only relatively short time scales, and the performance of the pseudo-sampling method was not obvious.

In Table 3, the rows denoted by *r* = “free” correspond with an assumption of free recombination (*i.e.*, evolving independently) among *R* + 1 loci. Rejection rates simulated by the exact binomial sampling and by the pseudosampling are given. Demographic Models 1 and 5 with reduced population sizes were used (see Table 3, legend). We did not observe any differences in the results generated by the binomial sampling *vs.* the pseudosampling under neutral or selective cases. These findings support the applicability of pseudo-sampling to our problem of concern.

**Table 3:** Results obtained by binomial sampling with various recombination fractions

*T*	*L*	Δ*t**^a^*	*r**^b^*	Neutral (*s*_0_ = 0)		Selective (*s*_0_ = 0.05)	
				Binomial*^c^*	Pseudo*^d^*	Binomial*^c^*	Pseudo*^d^*
[Model 1′]*^e^*							
10	2	5	Free	5.19	5.06	20.45	21.05
			0.1	4.66	−	19.90	−
			0.01	4.97	−	19.33	−
			0	5.14	−	19.85	−
20	5	5	Free	5.00	4.94	36.06	36.48
			0.1	4.97	−	36.05	−
			0.01	4.95	−	37.08	−
			0	4.84	−	35.76	−
[Model 5′]*^f^*							
5	1	5	Free	5.07	5.01	8.57	8.34
			0.1	4.93	−	8.80	−
			0.01	5.08	−	8.09	−
			0	5.41	−	8.13	−
10	5	2	Free	4.59	4.99	64.32	65.73
			0.1	5.27	−	64.23	−
			0.01	4.80	−	62.55	−
			0	4.98	−	63.21	−

Values indicate the rejection rates (%) obtained by 10,000 simulations for binomial sampling or by 100,000 simulations for pseudo-sampling under a nominal significance level of 5%.

The number of reference were *R* = 10 The initial frequencies for all *R* + 1 loci, *x_h_*_,0_, are assumed to be 0.5.

a $\Delta t=t_{i}-t_{i-1}\;\left(i=1,2,\ldots ,L\right)$.

b *r*, recombination fraction per generation between two adjacent loci of *R* + 1 loci. “Free” refers to free recombination.

c Binomial, the binomial sampling.

d Pseudo, the pseudo-sampling method used in this study.

e Model 1′, the constant-size model with *N* = 100.

f Model 5′, the severe bottleneck model with *N*(t) reduced to 1/200 of that in Model 5.

Next, we considered the case in which the reference loci and focal loci were not independent. We limited our analysis to the case in which *R* + 1 loci were in linkage equilibrium (LE) at t=0. For closely linked loci, linkage disequilibrium (LD) is a distinct possibility. In addition, selection at the focal locus can drastically promote LD (*e.g.*, Sabeti *et al.* 2002). However, for example, empirical studies of human genomes suggest that LD can be extended, at most, to several megabase pairs from the selective locus (*e.g.*, Saunders *et al.* 2005). Because the genome is large compared with the megabase pairs scale, we can select *R* reference loci such that *R* + 1 loci are in LE. For this reason, our discussion is limited to the case in which *R* + 1 loci are in LE.

In Table 3, the rows indicated by *r* = 0.1, 0.01, and 0 describe results corresponding to a case in which the per-generation recombination fraction between any two adjacent loci are 0.1, 0.01, and 0, respectively. At *t* = 0, *R* + 1 loci are assumed to be in LE. That is, the alleles at *R* + 1 loci are randomly combined to form haplotypes. The simulations were conducted by the exact binomial sampling. For the neutral or selective cases, we observed no obvious differences between free recombination and limited recombination (*r* = 0.1, 0.01, and 0; Table 3). That is, the type I error rates and powers were maintained regardless of recombination fractions.

**Figure 4:**
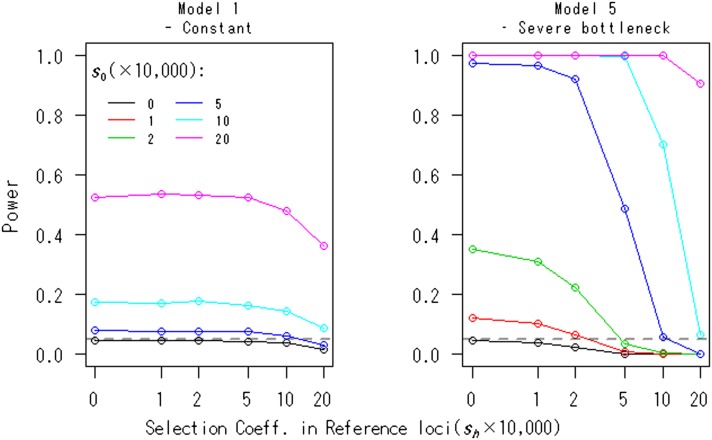
The effects of selection at reference loci on the power of the FITR. The powers of the FITR under various selection strengths, *s*_0_, at focal loci are shown as functions of the selection coefficient, $s_{h}(h\neq 0)$, at reference loci for demographic models 1 and 5. Each point corresponds to the power obtained by 100,000 simulations at the 5% significance level. The number of reference loci, the duration of sampling time, and the number of sampled points were *R* = 10, *T* = 1000, and (*L* + 1) = 11, respectively. The intervals between any two adjacent sampled points were the same at $\Delta t=t_{i}-t_{i-1}=100\;(i=1,2,\ldots ,L)$. The initial frequency for all R+1 loci, $x_{h,0}$, was assumed to be 0.5.

A case in which the reference loci are under selection is evaluated in Figure 4. The selection model is the same because the focal loci and *R* loci are under the same degree of selection. That is, for all h(≠0) loci, the fitnesses of genotypes *A_h_A_h_*, *A_h_a_h_*, and *a_h_a_h_* are assumed to be 1, 1 + 0.5*s_h_*, and 1 + *s_h_* (*s*_1_ = *s*_2_ = ··· = *s_R_*), respectively. The effects of selection at the reference loci are conservative for type I error rates (see the case of *s*_0_ = 0 in Figure 4). The results of Model 1 suggest that if $Ns_{h}<5$, there is little difference in rejection rates compared to the neutral case. Including Model 5, if the condition *s_h_* ≤ 1/2s_0_ is met, the power is not decreased. That is, the power is not highly sensitive to selection at the reference loci. Nevertheless, we recommend using synonymous sites or noncoding regions as references.

We next considered a situation in which allele frequencies at the reference loci were low at *t* = 0 and some allele frequencies could become 0 or 1 by *t* = *T*. When allele frequencies became 0 or 1, the statistic, *t*_FITR_, in (6) could not be defined. Therefore, it was practical to remove these loci from the calculation of *t*_FITR_. Table 4 indicates how rejection rates are changed by the data handling. Values in parentheses indicate the average number of reference loci used to calculate the FITR statistics. For *L* = 2 the type I error rate was inflated by a few percent (*e.g.*, 6.69% at most, Model 1) possibly because changes in allele frequencies at reference loci are biased toward smaller values when loci are removed for which the frequencies of alleles become 0 or 1. These apparently reduced changes in allele frequencies could bring about overestimates of change at the selective locus. For *L* = 10 the inflation of the type I error rate becomes small. In general, to prevent inflation of the type I error rate, loci having moderate frequencies of alleles (*e.g.*, ≥10%) should be used in this test.

**Table 4:** Effects of allele frequencies at the reference loci

*T*	*L*	Δ*t**^a^*	*x_h_*_,0_*^b^* (h ≠ 0)	Model 1		Model 5	
				Neutral (*s*_0_ = 0)	Selective (*s*_0_ = 0.002)	Neutral (*s*_0_ = 0)	Selective (*s*_0_ = 0.0005)
1000	2	500	0.5	4.86 (10.00)	50.03 (10.00)	5.05 (10.00)	82.93 (10.00)
			0.1	5.55 (9.84)	52.00 (9.84)	5.00 (10.00)	82.98 (10.00)
			0.05	6.69 (8.70)	53.11 (8.70)	5.28 (10.00)	82.85 (10.00)
			0.01	5.55 (3.37)	28.03 (3.37)	6.77 (7.84)	80.47 (7.84)
1000	10	100	0.5	4.91 (10.00)	53.49 (10.00)	4.93 (10.00)	97.44 (10.00)
			0.1	5.05 (9.84)	54.03 (9.85)	4.99 (10.00)	97.52 (10.00)
			0.05	5.18 (8.70)	53.95 (8.70)	4.99 (10.00)	97.34 (10.00)
			0.01	5.26 (3.38)	34.35 (3.38)	5.46 (7.84)	96.87 (7.83)

Values indicate rejection rates (%) obtained by 100,000 simulations under a nominal significance level of 5%. Values in parentheses correspond to the mean number of reference loci used to calculate the FITR statistics. The number of reference loci at *t*_0_ were *R* = 10. The initial frequency of the focal locus, *x*_0,0_, was assumed to be 0.5.

a $\Delta t=t_{i}-t_{i-1}\;\left(i=1,2,\ldots ,L\right)$.

b *x_h_*_,0_, the allele frequencies of the reference loci at *t*_0_.

**Figure 5:**
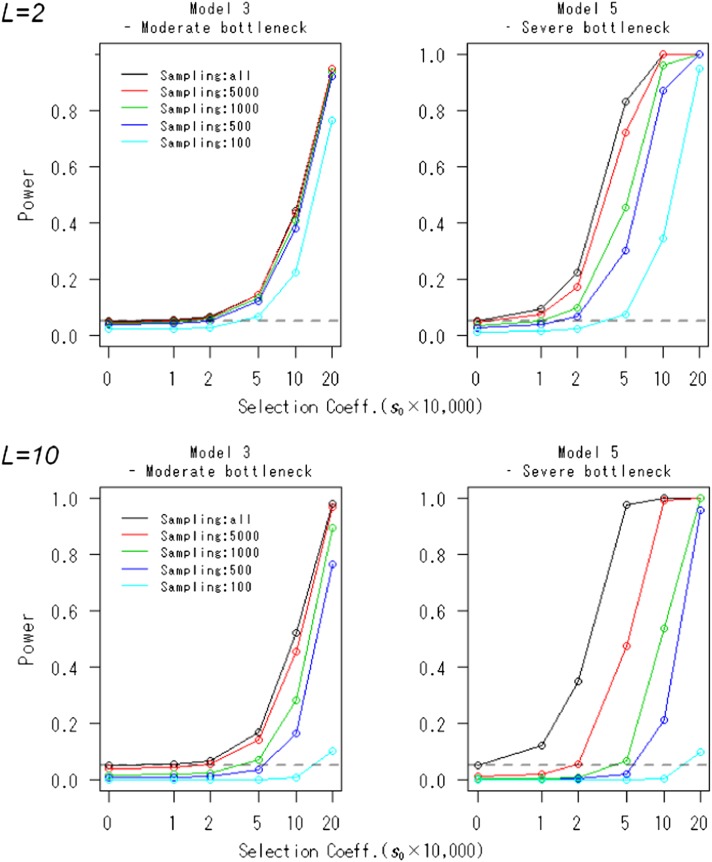
The effects of sampling error on the power of the FITR. The powers of the FITR under various sampling regimes are shown as functions of the selection coefficients for demographic models 3 and 5. All, or 5000, 1000, 500, or 100 individuals in a population were assumed to be sampled. Sampling was assumed to be conducted by binomial sampling at each (*R* + 1) locus and at each (*L* + 1) time point. Each point corresponds to the power obtained by 100,000 simulations at the 5% significance level. The number of reference loci, the duration of sampling time, and the number of sampled points were *R* = 10, *T* = 1000, and (*L* + 1) = 3 (upper graphs) or 11 (lower graphs), respectively. The intervals between any two adjacent sampled points were the same at Δ*_t_* = *t_i_–t_i-1_* = 500 (top graphs) or 100 (bottom graphs) (*i* = 1,2,…,L). The initial frequency for all R+1 loci, $x_{h,0}$, were assumed to be 0.5.

The effects of sampling error on the type I error rate and power of the FITR are shown in Figure 5. In general, the effects of sampling error on the type I error rate were conservative. As expected, the power decreased as the number of sampled individuals increased. The degree of power reduction differed for different demographic models or values of *L*. This finding reflects that the power is influenced by the relative magnitudes of changes in allele frequencies at *R* + 1 loci and sampling errors. As *L* increased, the relative changes in allele frequencies to the sampling errors decreased. Thus, power was more reduced for larger *L*. Regarding the demographic models, the population size of Model 5 was larger than that of Model 1. Therefore, the relative changes in allele frequencies to the sampling errors were larger in Model 5, and the degree of power is large in Model 5.

In this study, we proposed a neutrality test, the FITR, to accommodate fluctuations in population size using reference loci. Our test is an extension of Feder *et al.*’s (2013) FIT. By computer simulation, the actual type I error rate of the FITR was nearly equal to the nominal significant level regardless of fluctuations in population size when sampling noise could be ignored. The FITR detected selection with remarkable power under conditions of rapid growth (model 4) and severe bottleneck (model 5). Even under a model of constant population size, the FITR using 10 or more reference loci had more power than the FIT.

We also discussed the performance of the FITR in practical situations. The effects of selection at the reference loci were small unless selection was strong. Our findings indicated that when *R* + 1 were in LE, those loci should be considered independent of each other. In addition, loci with moderate frequencies of alleles should be used as references. Our findings may facilitate the development of more sophisticated methods using independent reference loci, including a method that can quantify (estimate) the strength of selection. These methods will enable appropriate inferences about natural selection in real and dynamic populations. Figure 5.

## Supplementary Material

Corrigendum

## Appendix

Suppose that there are two segregating alleles, denoted by *A_h_* and *a_h_*, at the *h*-th locus (h∈(0,1,2,…,R)). The fitnesses of genotypes *A_h_A_h_*, *A_h_a_h_*, and *a_h_a_h_* are assumed to be $1,\;1+0.5s_{h},\text{ and }1+s_{h}$, respectively. As described in the main text, the case of *h* = 0 corresponds to the focal locus. When $\Delta t_{i}=\left(t_{i}-t_{i-1}\right)$ is small compared with *N*_i_, the change in the allele frequency of $a_{h}$, $x_{h,i}-x_{h,i-1}$, from $t_{i-1}\;\text{to}\;t_{i}$ approximately follows a normal distribution,

$$x_{h,i}-x_{h,i-1}∼N\left(\frac{\Delta t_{i}}{2}s_{h}x_{h,i-1}(1-x_{h,i-1}),\;\frac{\Delta t_{i}x_{h,i-1}(1-x_{h,i-1})}{2N_{i}}\right).$$

The normalized change, $\Delta x_{h,i}=\left(x_{h,i}-x_{h,i-1}\right)/\sqrt{x_{h,i-1}\left(1-x_{h,i-1}\right)}$, is approximately

$$\Delta x_{h,i}∼N\left(\frac{\Delta t_{i}}{2}s_{h}\sqrt{x_{h,i-1}\left(1-x_{h,i-1}\right)},\;\frac{\Delta t_{i}}{2N_{i}}\right).$$

Then the probability density of $\Delta x_{h,i}$, $f_{h,i}\left(\Delta x_{h,i},s_{h},N_{i}\right)$, under $s_{h}\;\text{and }N_{i}$ is

$$f_{h,i}\left(\Delta x_{h,i},s_{h},N_{i}\right)\approx \sqrt{\frac{N_{i}}{\pi \Delta t_{i}}}exp\left(-\frac{N_{i}}{\Delta t_{i}}{\left(\Delta x_{h,i}-\frac{\Delta t_{i}}{2}s_{h}\sqrt{x_{h,i-1}(1-x_{h,i-1})}\right)}^{2}\right).$$

Considering $\Delta x_{h,i}$ for all h∈(0,1,2,…,R), the joint probability density of $\Delta x_{i}=(\Delta x_{0,i},\;\Delta x_{1,i}\cdots ,\Delta x_{R,i})$, $f_{i}\left(\Delta x_{i},s,N_{i}\right)$, under $s=\left(s_{0},s_{1},\cdots ,s_{R}\right)\;\text{and}\;N_{i}$.

$$f_{i}\left(\Delta x_{i},s,N_{i}\right)\approx {\left(\frac{N_{i}}{\pi \Delta t_{i}}\right)}^{\frac{R+1}{2}}exp\left(-\frac{N_{i}}{\Delta t_{i}}\sum_{h=0}^{R}{\left(\Delta x_{h,i}-\frac{\Delta t_{i}}{2}s_{h}\sqrt{x_{h,i-1}(1-x_{h,i-1})}\right)}^{2}\right).$$ (A1)

i) $t_{FITR(i)}$ **is the exact LRS using data** $\Delta x_{i}$
  We will show that $t_{FITR(i)}$ given by (3) or (4) is the exact statistic using data $\Delta x_{i}$ to test $H_{0}:s_{h}=0$ (for all *h*) against $H_{1}:s_{0}\neq 0$ and $s_{h}=0$ (h≠0) in the likelihood ratio test framework.
  In our model, the likelihood ratio test is a test for which we reject $H_{0}$ if
  $$\lambda =\frac{f_{i}\left(\Delta x_{i},0,{\overset{ˇ}{N}}_{i}\right)}{f_{i}\left(\Delta x_{i},\left({\hat{s}}_{0},0\right),{\hat{N}}_{i}\right)}<c\;(constant).$$ (A2)
  Otherwise, we accept *H*_0_. Here, ${\overset{ˇ}{N}}_{i}$ is the maximum likelihood estimator (MLE) of *N_i_* under *H*_0_, and ${\hat{N}}_{i}$ and ${\hat{s}}_{0}$ are the MLEs of $N_{i}$ and $s_{0}$ under *H*_1_.
  Under *H*_0_, the probability density of $\Delta x_{h,i}$ is given by
  $$f_{i}\left(\Delta x_{i},0,N_{i}\right)={\left(\frac{N_{i}}{\pi \Delta t_{i}}\right)}^{\frac{R+1}{2}}exp\left(-\frac{N_{i}}{\Delta t_{i}}\sum_{h=0}^{R}\Delta x_{h,i}^{2}\right)$$
  from (A1). Solving
  $$\frac{\partial \text{ln}f_{i}}{\partial N_{i}}=\frac{R+1}{2N_{i}}-\frac{1}{\Delta t_{i}}\sum_{h=0}^{R}\Delta x_{h,i}^{2}=0,$$
  we get
  $${\overset{ˇ}{N}}_{i}=\frac{(R+1)\Delta t_{i}}{2\sum_{h=0}^{R}\Delta x_{h,i}^{2}}.$$
  Then
  $$f_{i}\left(\Delta x_{i},0,{\overset{ˇ}{N}}_{i}\right)={\left(\frac{{\overset{ˇ}{N}}_{i}}{\text{π}\Delta t_{i}}\right)}^{\frac{R+1}{2}}\text{exp}\left(-\frac{R+1}{2}\right)$$
  Under *H*_1_, the probability density of $\Delta x_{h,i}$ is given by
  $$f_{i}\left(\Delta x_{i},(s_{0},0),N_{i}\right)={\left(\frac{N_{i}}{\pi \Delta t_{i}}\right)}^{\frac{R+1}{2}}exp\left(-\frac{N_{i}}{\Delta t_{i}}{\left(\Delta x_{0,i}-\frac{\Delta t_{i}}{2}s_{0}\sqrt{x_{0,i-1}(1-x_{0,i-1})}\right)}^{2}-\frac{N_{i}}{\Delta t_{i}}\sum_{h=1}^{R}\Delta x_{h,i}^{2}\right)$$
  from (A1). Solving
  $$\frac{\partial \text{ln}f_{i}}{\partial N_{i}}=\frac{R+1}{2N_{i}}-\frac{1}{\Delta t_{i}}{\left(\Delta x_{0,i}-\frac{\Delta t_{i}}{2}s_{0}\sqrt{x_{0,i-1}(1-x_{0,i-1})}\right)}^{2}-\frac{1}{\Delta t_{i}}\sum_{h=1}^{R}\Delta x_{h,i}^{2}=0\;\text{and}$$
  $$\frac{\partial \text{ln}f_{i}}{\partial s_{0}}=N_{i}\left(\Delta x_{0,i}\sqrt{x_{0,i-1}(1-x_{0,i-1})}-\frac{\Delta t_{i}}{2}s_{0}x_{0,i-1}(1-x_{0,i-1})\right)=0,$$
  we get
  $${\hat{N}}_{i}=\frac{(R+1)\Delta t_{i}}{2\sum_{h=1}^{R}\Delta x_{h,i}^{2}}\;\text{and}$$
  $${\hat{s}}_{0}=\frac{2\Delta x_{0,i}}{\Delta t_{i}\sqrt{x_{0,i-1}(1-x_{0,i-1})}}.$$
  Then
  $$f_{i}\left(\Delta x_{i},\left({\hat{s}}_{0},0\right),{\hat{N}}_{i}\right)={\left(\frac{{\hat{N}}_{i}}{\text{π}\Delta t_{i}}\right)}^{\frac{R+1}{2}}\exp\left(-\frac{R+1}{2}\right).$$
  Thus, (A2) is equivalent to
  λ=fi(Δxi,0,Nˇi)fi(Δxi,(s^0,0),N^i)=(NˇiN^i)R+12=(1∑h=0RΔxh,i21∑h=1RΔxh,i2)R+12=(∑h=0RΔxh,i2∑h=1RΔxh,i2)− R+12=(1+Δx0,i2∑h=1RΔxh,i2)−R+12<c.
  With some algebra, we let a new constant $c^{\prime }=Rc^{-\frac{2}{R+1}}-R$
  $$\frac{\Delta x_{0,i}^{2}}{\frac{1}{R}\sum_{h=1}^{R}\Delta x_{h,i}^{2}}>c^{\prime }.$$
  Finally, we get
  $$\frac{\Delta x_{0,i}}{\sqrt{\frac{1}{R}\sum_{h=1}^{R}\Delta x_{h,i}^{2}}}=t_{FITR(i)}<-c^{\prime \prime },\;\;c^{\prime \prime }<\frac{\Delta x_{0,i}}{\sqrt{\frac{1}{R}\sum_{h=1}^{R}\Delta x_{h,i}^{2}}}=t_{FITR\left(i\right)}$$
  where $c^{\prime \prime }=\sqrt{c^{\prime }}$. Therefore, *t_FITR(i)_* as given by (3) or (4) is the exact LRS using data $\Delta x_{i}$.
ii) **Ad hoc interpretation for *t_FITR_* as a test statistic using the data Δ*x***
  As *R* grows, the *t* distribution for *t_FITR(i)_* approaches the normal distribution with mean $s_{0}\sqrt{N_{i}\Delta t_{i}x_{0,i-1}(1-x_{0,i-1})/2}$ and variance 1,
  $$t_{FITR\left(i\right)}=\frac{Y_{0,i}}{\sqrt{\frac{1}{R}\sum_{h=1}^{R}Y_{h,i}^{2}}}∼N\left(s_{0}\sqrt{N_{i}\Delta t_{i}x_{0,i-1}\left(1-x_{0,i-1}\right)/2},\;1\right).$$
  Unfortunately, $s_{0}\sqrt{N_{i}\Delta t_{i}x_{0,i-1}(1-x_{0,i-1})/2}$ varies with *N*_i_, $\Delta t_{i}$, and $x_{0,i-1}$. When $x_{0,i-1}=0.5,\;0.4,\;0.3,0.2,\;0.1,\text{ and }0.05$; $\sqrt{x_{0,i-1}(1-x_{0,i-1})}=$ 0.50, 0.490, 0.458, 0.400, 0.300 and 0.218, respectively. Nevertheless, the values of $\sqrt{x_{0,i-1}(1-x_{0,i-1})}$ are roughly the same if $x_{0,i-1}$ are far from 0 or 1. In addition, the values of $\sqrt{N_{i}}$ and $\sqrt{\Delta t_{i}}$ vary with *i*. However, we try to consider $s_{0}\sqrt{N_{i}\Delta t_{i}x_{0,i-1}(1-x_{0,i-1})/2}$ as a constant *μ*, such that
  $$t_{FITR(i)}∼N\left(\mu ,1\right).$$
  Consider a test *H*_0_:μ = 0 against *H*_1_:μ ≠ 0 using *L* i.i.d samples from the distribution. The LRS is given by the sum of $t_{FITR(i)}$, $\sum_{i=1}^{L}t_{FITR(i)}$. That is, when *R* is large and the variation of the value for $\sqrt{N_{i}\Delta t_{i}x_{0,i-1}(1-x_{0,i-1})}$ is not large, *t_FITR_* given by (5) or (6) is expected to be close to the LRS.

## References

[bib1] Bollback J. P. Huelsenbeck J. P., 2007 Clonal interference is alleviated by high mutation rates in large populations. Mol. Biol. Evol. 24: 1397–1406. 17379621 10.1093/molbev/msm056

[bib2] Bollback J. P. York T. L. Nielsen R., 2008 Estimation of 2Nes from temporal allele frequency data. Genetics 179: 497–502. 18493066 10.1534/genetics.107.085019 PMC2390626

[bib3] Feder A. Kryazhimskiy S. Plotkin J. B., 2014 Identifying signatures of selection in genetic time series. Genetics 196: 509–522. 24318534 10.1534/genetics.113.158220 PMC3914623

[bib4] Fisher R. A., 1925 Applications of “Student’s” distribution. Metron 5: 90–104.

[bib5] Gallet R. Cooper T. F. Elena S. F. Lenormand T., 2012 Measuring selection coefficients below 10^−3^: method, questions, and prospects. Genetics 190: 175–186. 22042578 10.1534/genetics.111.133454 PMC3249376

[bib6] Hummel S. Schmidt D. Kremeyer B. Herrmann B. Oppermann M., 2005 Detection of the CCR5–D32 HIV resistance gene in Bronze Age skeletons. Genes Immun. 6: 371–374. 15815693 10.1038/sj.gene.6364172

[bib7] Illingworth C. J. R. Mustonen V., 2011 Distinguishing driver and passenger mutations in an evolutionary history categorized by interference. Genetics 189: 989–1000. 21900272 10.1534/genetics.111.133975 PMC3213378

[bib8] Illingworth C. J. Parts L. Schiffels S. Liti G. Mustonen V., 2012 Quantifying selection acting on a complex trait using allele frequency time series data. Mol. Biol. Evol. 29: 1187–1197. 22114362 10.1093/molbev/msr289 PMC3731369

[bib9] Kimura M., 1980 Average time to fixation of a mutant allele in a finite population under continued mutation pressure: studies by analytical, numerical and pseudosampling methods. Proc. Natl. Acad. Sci. USA 77: 522–526. 16592764 10.1073/pnas.77.1.522 PMC348304

[bib10] Kimura M. Takahata N., 1983 Selective constraint in protein polymorphism: study of the effectively neutral mutation model by using an improved pseudosampling method. Proc. Natl. Acad. Sci. USA 80: 1048–1052. 6573657 10.1073/pnas.80.4.1048 PMC393525

[bib11] Lehmann E. L. Romano J. P., 2005 Testing Statistical Hypotheses, Ed. 3 Springer, New York.

[bib12] Malaspinas A. S. Malaspinas O. Evans S. N. Slatkin M., 2012 Estimating allele age and selection coefficient from time-serial data. Genetics 192: 599–607. 22851647 10.1534/genetics.112.140939 PMC3454883

[bib13] Mathieson I. McVean G., 2013 Estimating selection coefficients in spatially structured populations from time series data of allele frequencies. Genetics 193: 973–984. 23307902 10.1534/genetics.112.147611 PMC3584010

[bib14] Pawitan Y., 2001 In All Likelihood: Statistical Modeling and Inference Using Likelihood, Oxford University Press, New York.

[bib15] Saunders M. Slatkin M. Garner C. Hammer M. Nachman M., 2005 The extent of linkage disequilibrium caused by selection on G6PD in humans. Genetics 171: 1219–1229. 16020776 10.1534/genetics.105.048140 PMC1456824

[bib16] Sabeti P. C. Reich D. E. Higgins J. M. Levine H. Z. P. Richter D. J., 2002 Detecting recent positive selection in the human genome from haplotype structure. Nature 419: 832–837. 12397357 10.1038/nature01140

