# Supplementary material for: Detecting Selection Using Time-Series Data of Allele Frequencies with Multiple Independent Reference Loci
Source: G3 (Bethesda). 2013 Sep 30;3(12):2151–61. doi: 10.1534/g3.113.008276 (PMC3852378; doi:10.1534/g3.113.008276)
Supplement: Corrigendum [file supp_3_12_2151__index.html]

Corrigendum 

# Detecting Selection Using Time-Series Data of Allele Frequencies with Multiple Independent Reference Loci

## Corrigendum for Nishino, G3: Genes|Genomes|Genetics 3 (12) 2151-2161.

**Files in this Data Supplement:**

- Corrigendum - Corrigendum for Nishino, G3: Genes|Genomes|Genetics 3 (12) 2151-2161.
