# Supplementary material for: Detecting Selection Using Time-Series Data of Allele Frequencies with Multiple Independent Reference Loci
Source: G3 (Bethesda). 2013 Sep 30;3(12):2151–61. doi: 10.1534/g3.113.008276 (PMC3852378; doi:10.1534/g3.113.008276)
Supplement: Corrigendum [file supp_g3.113.008276_Corrigendum_for_Nishino.pdf]

Corrigendum for Nishino, *G3: Genes|Genomes|Genetics* 3 (12) 2151-2161.

G3: Genes|Genomes|Genetics, Vol 3, 2151-2161, December 2013, Copyright © 2013 Nishino.

CORRIGENDUM

In the article by J. Nishino, (*G3: Genes|Genomes|Genetics* 3: 2151-2161) entitled “Detecting Selection Using Time-Series Data of Allele Frequencies with Multiple Independent Reference Loci” the citation information for Feder *et al*. has been updated to read:

Feder, A. F., S. Kryazhimskiy, and J. B. Plotkin, 2014. Identifying Signatures of Selection in Genetic Time Series. GENETICS 196:509-522.
